# Loss of glutathione homeostasis associated with neuronal senescence facilitates TRPM2 channel activation in cultured hippocampal pyramidal neurons

**DOI:** 10.1186/1756-6606-5-11

**Published:** 2012-04-09

**Authors:** Jillian C Belrose, Yu-Feng Xie, Lynn J Gierszewski, John F MacDonald, Michael F Jackson

**Affiliations:** 1 Department of Anatomy and Cell Biology, University of Western Ontario, London, ON, N6A 5 K8, Canada; 2 Robarts Research Institute, Molecular Brain Research Group, University of Western Ontario, 100 Perth Drive, London, ON, N6A 5 K8, Canada; 3 Department of Physiology and Pharmacology, University of Western Ontario, London, ON, N6A 5 K8, Canada; 4 Robarts Research Institute, Molecular Brain Research Group, Department of Physiology and Pharmacology, University of Western Ontario, 100 Perth Drive, London, ON, N6A 5 K8, Canada

**Keywords:** TRPM2, Aging, Glutathione, Oxidative stress, Pyramidal neuron, Primary hippocampal culture

## Abstract

**Background:**

Glutathione (GSH) plays an important role in neuronal oxidant defence. Depletion of cellular GSH is observed in neurodegenerative diseases and thereby contributes to the associated oxidative stress and Ca^2+ ^dysregulation. Whether depletion of cellular GSH, associated with neuronal senescence, directly influences Ca^2+ ^permeation pathways is not known. Transient receptor potential melastatin type 2 (TRPM2) is a Ca^2+ ^permeable non-selective cation channel expressed in several cell types including hippocampal pyramidal neurons. Moreover, activation of TRPM2 during oxidative stress has been linked to cell death. Importantly, GSH has been reported to inhibit TRPM2 channels, suggesting they may directly contribute to Ca^2+ ^dysregulation associated with neuronal senescence. Herein, we explore the relation between cellular GSH and TRPM2 channel activity in long-term cultures of hippocampal neurons.

**Results:**

In whole-cell voltage-clamp recordings, we observe that TRPM2 current density increases in cultured pyramidal neurons over time in vitro. The observed increase in current density was prevented by treatment with NAC, a precursor to GSH synthesis. Conversely, treatment of cultures maintained for 2 weeks in vitro with L-BSO, which depletes GSH by inhibiting its synthesis, augments TRPM2 currents. Additionally, we demonstrate that GSH inhibits TRPM2 currents through a thiol-independent mechanism, and produces a 3.5-fold shift in the dose-response curve generated by ADPR, the intracellular agonist for TRPM2.

**Conclusion:**

These results indicate that GSH plays a physiologically relevant role in the regulation of TRPM2 currents in hippocampal pyramidal neurons. This interaction may play an important role in aging and neurological diseases associated with depletion of GSH.

## Introduction

Aging is a major risk factor for developing several neurodegenerative diseases. Although the precise reasons are poorly understood, a growing body of evidence suggests that age-related cognitive decline is associated with stereotypic changes in cellular homeostasis which ultimately lead to impaired neuronal function [1]. A leading hypothesis suggests that increased oxidative stress associated with aging predisposes neurons to dysregulated intracellular Ca^2+ ^homeostasis in response to disease causing factors [2]. Accordingly, understanding the relation between oxidative stress and altered Ca^2+ ^homeostasis may provide valuable insight into mechanisms underlying neurodegenerative disease.

Glutathione (GSH), the most abundant intracellular reducing agent [3], plays an important role in limiting cellular damage by reactive oxygen and nitrogen species generated as a by-product of aerobic metabolism. Cellular levels of GSH are known to decrease with age [4-9] and the resulting decline in antioxidant defence is viewed as a risk factor contributing to the increased susceptibility to neurodegenerative disease associated with aging. For example, a strong relationship has been demonstrated between reduced levels of GSH and Parkinson's disease [10-16]. Furthermore, a reduction in glutathione has also been associated with cerebral ischemia [13-16]. Importantly, depletion of GSH itself leads to increased oxidative stress, altered Ca^2+ ^homeostasis and increased neuronal cell death [17-20]. It is unknown whether depletion of GSH promotes Ca^2+ ^dysregulation directly (e.g. by altering Ca^2+ ^permeation pathways) or through the induction of oxidative stress.

TRPM2 is a highly unique calcium permeable non-selective cation channel that is responsive to reactive oxygen and nitrogen species (ROS/RNS). Channel activation by ROS/RNS is proposed to occur following the generation of adenosine diphosphate ribose (ADPR), which serves as an intracellular agonist [21]. Notably, gating is also greatly facilitated by elevated concentrations of intracellular Ca^2+ ^[22,23]. TRPM2 expression is highest within the brain [24] and recent work in our lab demonstrated that TRPM2 is expressed and functional in pyramidal neurons of the hippocampus [23], a subset of neurons that are particularly susceptible to oxidative stress-induced damage in stroke and neurodegenerative disease [25,26]. This current was absent in primary hippocampal neurons cultured from TRPM2 knockout (KO) animals [27]. Importantly, TRPM2 has been shown to contribute to cell death in response to oxidative stress, TNFα, amyloid-β peptide, and ischemia [28-30]. These findings raise the possibility that TRPM2 may play an important role in neurological diseases associated with aging, excitotoxicity and oxidative stress.

Recent evidence suggests that TRPM2 channels may be directly regulated by GSH. Indeed, GSH has been shown to inhibit TRPM2 currents in astrocytes, microglia, and dorsal root ganglion sensory neurons [20,31]. Importantly, these papers did not investigate the effects of glutathione on TRPM2 channels in neurons susceptible to degeneration. Furthermore, the mechanism responsible for the observed inhibition was not explored.

We now extend previous findings demonstrating GSH-mediated inhibition of TRPM2 currents by demonstrating that intrinsic, age-dependent, variations in the intracellular concentration of GSH is a key determinant in dictating the level of TRPM2 activation in neurons. Moreover, we demonstrate that the inhibition of TRPM2 function by GSH operates independently of its capacity as a reducing agent. These findings suggest that altered intracellular Ca^2+ ^dynamics associated with GSH depletion, induced experimentally or with aging, may develop as a result of altered TRPM2 regulation.

## Results

### TRPM2 currents increase with time in vitro

Primary neuronal cultures are often used to examine neuronal development; however, beyond 21 days in vitro, early neuronal markers such as nestin drastically diminishes in expression, and mature neuronal markers such as MAP-2 are expressed, indicative of a mature neuronal population [32]. Furthermore, cultures maintained beyond 3 weeks in vitro recapitulate many of the characteristic cellular changes that have been associated with aging and neurodegeneration [32-35]. These include increased oxidative stress, lipid peroxidation, protein tyrosine nitration, mitochondrial injury, ER stress, DNA damage, Ca^2+ ^dysregulation, calpain cleavage, endogenous Aβ accumulation, and susceptibility to neuronal cell death. Importantly, in primary neuronal cultures the concentration of GSH has also been shown to decrease with time in vitro [36,37]. Accordingly, long-term neuronal cultures serve as a valuable model system for the study of cellular changes associated with neuronal aging and senescence. With this in mind, we first sought to determine whether in vitro neuronal senescence correlates with altered TRPM2 function.

**Figure 1:**
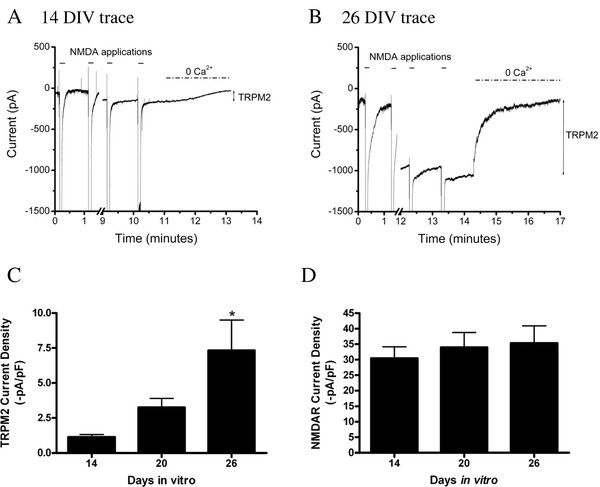
**TRPM2 currents are enhanced in hippocampal pyramidal neurons with time in vitro**. With 1 mM ADPR included in the patch pipette, TRPM2 currents developed slowly following repeated (1/60 sec) applications NMDA (100 μM, for 5 sec). TRPM2 currents were quantified by removing extracellular calcium and amplitude was normalized to cell capacitance. NMDA applications are indicated with solid lines. Calcium-free application is depicted as a dashed line (**A**) Representative trace of TRPM2 currents recorded at 14 DIV (**B**) Representative trace of TRPM2 currents recorded at 25 DIV. (**C**) Current density at 14, 20, and 26 DIV (n = 5 per group) were compared by one-way ANOVA followed by *post-hoc *Tukey's test. A significant increase in TRPM2 current density between 14 and 26 DIV was observed (p < 0.05). (**D**) NMDAR current density is not significantly increased over time in vitro (*p *= 0.76).

To rigorously examine TRPM2 function over time in culture, whole-cell voltage-clamp recordings were made from primary hippocampal neuronal cultures maintained in vitro for up to 28 days. Using a previously established protocol [23], TRPM2 currents were evoked by intracellular administration of ADPR paired with repeated N-methyl-D-aspartate (NMDA) receptor (NMDAR) stimulation. Importantly, the currents generated using this protocol can be entirely attributed to TRPM2 channels as they are abolished by known blockers of TRPM2 [23]. To ensure that the full complement of TRPM2 channels expressed at various time points are being activated in our recordings, a saturating concentration of ADPR (1 mM) was included in the patch pipette. Furthermore, as the neuronal cell surface area increases over time due to the continued extension of neuronal processes, TRPM2 currents were normalized to membrane capacitance, measurement of which correlates with cell surface area (*p *< 0.05, data not shown). As illustrated by the representative traces shown in Figure 1A and 1B, repeated applications of NMDA to pyramidal neurons loaded with ADPR caused the progressive development of large inward currents, the amplitudes of which stabilized within approximately 5-15 min. The amplitude of TRPM2 currents was then determined after removal of extracellular Ca^2+^, a protocol known to abolish TRPM2-mediated currents [23,27]. Recordings were made from neurons at 14, 20, and 26 days in vitro (DIV, n = 5/group). Results demonstrate that there is an increase in TRPM2 current density between 14 and 26 DIV in cultured hippocampal pyramidal neurons (Figure 1C, *p *< 0.05). Since activation of the NMDAR was used to elicit the TRPM2 current, we also analyzed whether the NMDAR current density is increased over time in vitro. No change was observed over this same time period (*p *= 0.76, Figure 1D). Furthermore, we assessed whether a change in NMDAR kinetics may explain the enhanced TRPM2 response by calculating the integral under the NMDAR application and normalized this measurement to capacitance (Q/pF). Similarly, no change was observed from 2 - 4 weeks in vitro (data not shown, *p *= 0.39).

### TRPM2 mRNA does not change with time in vitro

**Figure 2:**
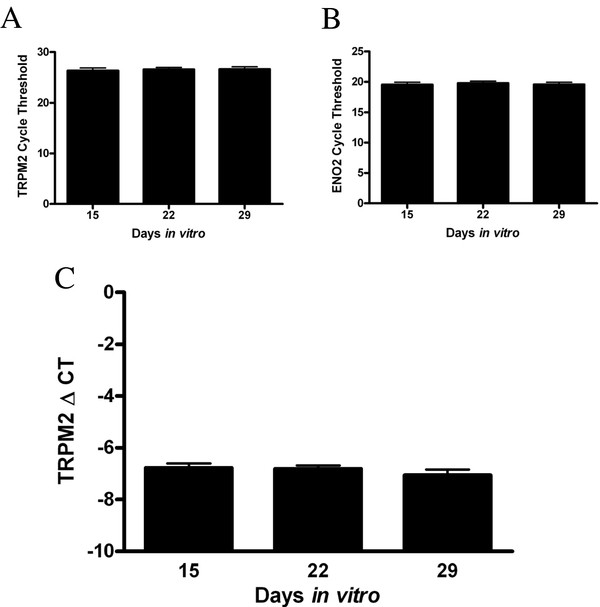
**TRPM2 mRNA levels do not change in hippocampal primary cultures with time in vitro**. (**A**) TRPM2 mRNA was measured at 14, 21, and 29 DIV (n = 8 per group) by quantitative real-time PCR (qPCR). No significant change in cycle threshold values is observed (*p *= 0.89) (**B**) QPCR analysis of neuron specific enolase (ENO2) mRNA values used to control for neuronal content over time. No significant difference was observed (*p *= 0.9). (**C**) Normalized TRPM2 values for each sample (ENO2-TRPM2) were quantified. No difference in the normalized levels of TRPM2 mRNA is seen over time in culture (*p *= 0.51).

Increased TRPM2 current density with time in vitro could be attributed to an increase in the function of TRPM2, potentially via a decrease in glutathione, or alternatively an increase in TRPM2 expression. Using real-time quantitative PCR (RT-QPCR), we tested whether TRPM2 mRNA increases with time in vitro in primary hippocampal cell culture. Total RNA from cells at 15 (n = 9), 22 (n = 8), and 29 (n = 9) DIV were run in duplicate and TRPM2 levels were quantified. Results were normalized to neuron-specific enolase (Eno2) by calculating delta cycle threshold (ΔCT) values for each sample (NSE CT - TRPM2 CT) in order to control for any variability in neuronal content over time. Analysis by one-way ANOVA revealed no change in the TRPM2 cycle threshold (*p *= 0.89, Figure 2A), Eno2 (*p *= 0.9, Figure 2B) or normalized TRPM2 mRNA levels over time in vitro (*p *= 0.51, Figure 2C). These findings demonstrate that changes in mRNA expression levels are not responsible for the increase in TRPM2 current density observed over time in vitro.

### Modulation of glutathione alters TRPM2 currents in hippocampal pyramidal neurons

If decreased cellular GSH content is responsible for elevated TRPM2 function over time in vitro then enhancing intracellular GSH levels should diminish TRPM2 current amplitude in older cultured neurons (~28 DIV). Conversely, reducing intracellular GSH levels should facilitate TRPM2 channel function in younger neuronal cultures (~14 DIV).

**Figure 3:**
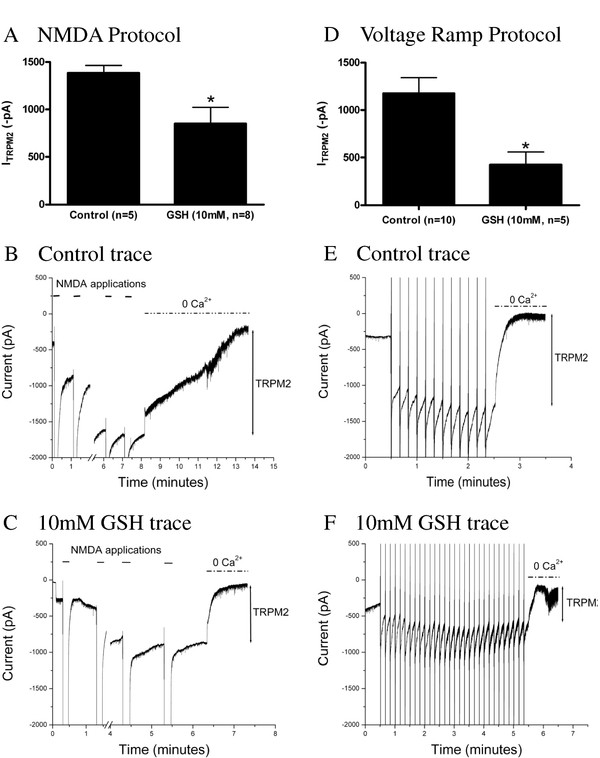
**Glutathione inhibits TRPM2 currents in hippocampal pyramidal neurons**. (**A**) 10 mM GSH in the patch pipette significantly inhibits TRPM2 currents generated by 1 mM ADPR with an NMDAR stimulation protocol (*p *= 0.04). (**B**, **C**) Representative traces of control and GSH inhibition with the NMDAR stimulation protocol. (**D**)10 mM GSH in the patch pipette significantly inhibits TRPM2 currents generated by 1 mM ADPR with a voltage ramp protocol (*p *= 0.01). (**E**,**F**) Representative traces of control and GSH inhibition with the voltage ramp protocol.

We first examined the consequence of elevating the intracellular concentration of GSH in neurons at 4 weeks in vitro by exogenous application through the patch pipette. When compared with controls, GSH (10 mM) significantly inhibited TRPM2 currents activated in the presence of ADPR by the NMDAR stimulation protocol (Figure 3A, *p *= 0.04). In millimolar concentrations, extracellular GSH has been shown to enhance NMDAR function [38,39]. No difference in NMDAR amplitude was observed with intracellular delivery of GSH (*p *= 0.82, data not shown). Representative traces are shown in Figure 3B (control) and Figure 3C (10 mM GSH). To confirm the effects of GSH on TRPM2 currents, we also examined the effects of GSH delivered through the patch pipette on TRPM2 currents generated by a voltage ramp protocol. Using this alternative protocol, GSH similarly inhibited TRPM2 currents (Figure 3D, *p *= 0.01). Examples of representative traces for control and GSH voltage ramp recordings are shown in Figure 3E and 3F, respectively.

**Figure 4:**
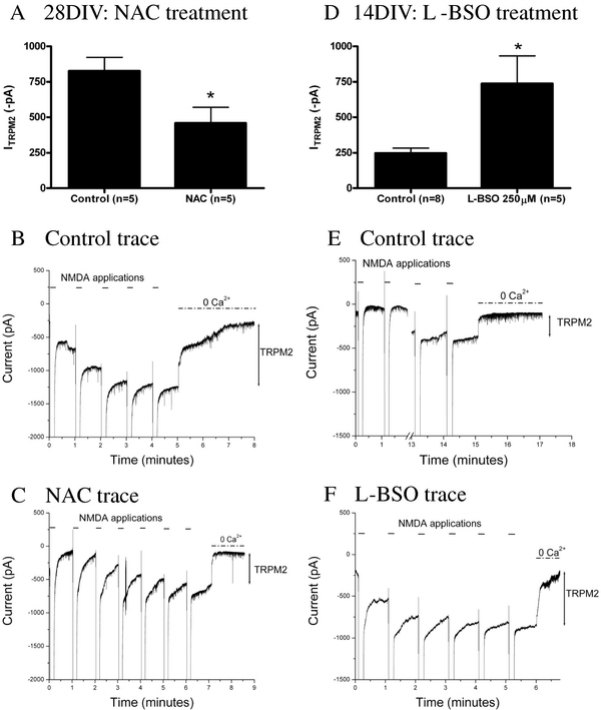
**(**A**) Treatment from 1-4 weeks in vitro with 50 μM NAC significantly inhibited TRPM2 currents generated by 0.3 mM ADPR and an NMDAR stimulation protocol in cultures at 28 DIV (*p *= 0.04)**. (**B**, **C**) Representative control and NAC traces. (**D**) A 48 hour treatment with 250 μM L-BSO significantly enhances TRPM2 currents generated with 1 mM ADPR with an NMDAR stimulation protocol in hippocampal pyramidal neurons at 2 weeks in vitro. (**E**, **F**) Representative control and L-BSO traces.

We next sought evidence that augmenting endogenous GSH content can similarly decrease TRPM2 function. We chronically treated neurons from 1-4 weeks in vitro with N-acetyl-cysteine (NAC), a precursor to GSH that has been shown to increase levels of endogenous GSH [14,40-42] and examined whether TRPM2 currents in cultures at 4 weeks in vitro were reduced. Treatment with 50 μM NAC beginning at 1 week in vitro significantly attenuated TRPM2 currents in cultures at 28 DIV (*p *= 0.04, Figure 4A-C). Previous studies have shown that NAC is able to act as a reactive oxygen scavenger independent of its ability to upregulate GSH [43]. To confirm that the observed effect can be attributed to GSH and not to NAC itself, NAC (50 μM) was included in the patch pipette and TRPM2 currents were elicited with the NMDA activation protocol with 1 mM ADPR in the patch pipette. Compared to controls (1076 ± 204.9, n = 3), acute NAC (910.5 ± 142.8 n = 4) did not alter TRPM2 currents (*p *= 0.52, data not shown). These results confirm previous findings that GSH can inhibit TRPM2 currents, and are consistent with the notion that reduced GSH, at least in part, may underlie the increase in TRPM2 currents that we observed in hippocampal pyramidal neurons over time in vitro. These findings also imply that GSH constitutively suppresses TRPM2 function in younger cultured neurons.

Accordingly, we next examined whether depleting GSH could augment TRPM2 function in young cultures maintained for 2 weeks in vitro. L-buthionine-(S, R)-sulfoximine (L-BSO) inhibits the enzyme γ-glutamylcysteine synthetase, the rate limiting step in GSH synthesis [44] and is effective in depleting GSH [14,18,36,42,45]. Consistent with our hypothesis, TRPM2 currents were significantly potentiated, when compared with controls (*p *= 0.01), in 2 week old cultured neurons depleted of GSH by treatment with 250 μM L-BSO for 48 hours (Figure 4D-F).

### Mechanism of TRPM2 inhibition by GSH

**Figure 5:**
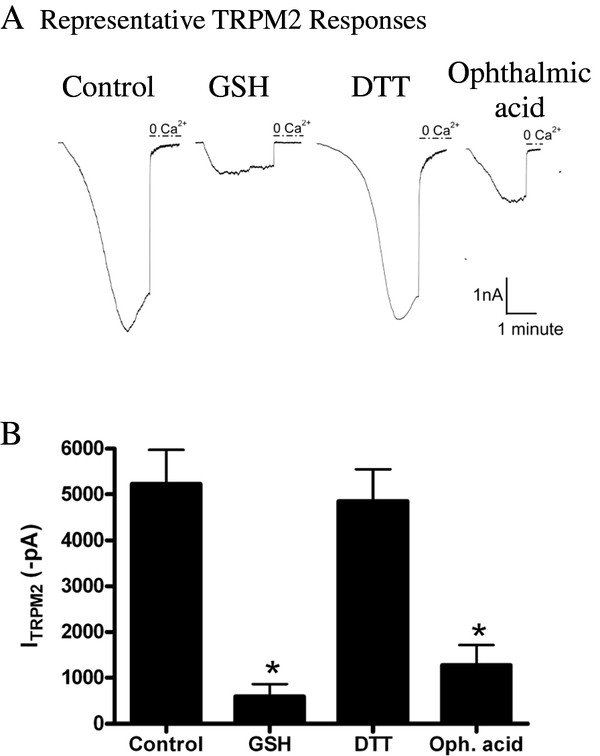
**GSH inhibits TRPM2 in a thiol-independent mechanism**. TRPM2 currents were elicited in HEK293-TRPM2 cells using a 2 mM calcium extracellular solution and 0.1 mM ADPR. TRPM2 currents were then inhibited and quantified following removal of extracellular calcium (**A**) Representative TRPM2 responses obtained in control (n = 12), GSH (n = 10), DTT (n = 6) and ophthalmic acid (n = 6) groups (**B**) TRPM2 currents are significantly inhibited with 10 mM GSH or 10 mM ophthalmic acid, but are not altered when 10 mM DTT is included in the patch pipette (One-way ANOVA followed by *post-hoc *Tukey's test, * *p *< 0.05).

HEK 293 cells stably expressing TRPM2 under control of an inducible promoter (TRPM2-HEK293) were used to characterize the mechanism of glutathione-mediated TRPM2 inhibition. To further validate our findings in hippocampal pyramidal neurons, and previous findings in glial and dorsal root ganglion cells [20,31], TRPM2 currents from TRPM2-HEK293 cells were recorded in the whole-cell patch clamp configuration with 0.1 mM ADPR in the patch pipette. Of note, HEK293 cells do not express NMDARs nor voltage-gated Ca^2+ ^channels. However, intracellular application of ADPR alone is sufficient to activate TRPM2 channels in this model system, presumably due to the much higher induced channel expression. Currents generated under control conditions were compared to those generated when 10 mM GSH was included in the patch pipette, and removal of extracellular Ca^2+ ^was used to abolish the TRPM2 current. In the presence of exogenously delivered intracellular GSH, a substantial attenuation of the TRPM2 current was observed (Figure 5, p < 0.001).

GSH may regulate channel function through a number of distinct mechanisms including redox mechanisms as well as post-translational modification involving the formation mixed protein disulfides (i.e. glutathionylation). For example, through its reducing potential GSH has been shown to regulate the function of other receptors and channels, including NMDARs [38,46]. To examine the possibility that TRPM2 is regulated through changes in its redox state, we examined whether the inhibitory effect of GSH could be mimicked by another reducing agent, dithiothreitol (DTT). DTT was included in the patch pipette and currents were recorded from TRPM2-HEK293 cells. At 10 mM DTT, no change in TRPM2 amplitude was observed when compared to controls (*p *> 0.05; Figure 5), suggesting that the reducing ability of GSH is not responsible for the change in TRPM2 currents.

GSH has also been shown to inhibit currents through direct binding to channels, either at thiol residues, known as glutathionylation, or at other non-thiol containing binding sites [47-49]. Ophthalmic acid (γ-glutamyl-2-amino-*n*-butanoylglycine) is an analogue of GSH which contains L-2-aminobutyrate in place of the cysteine residue [50]. This analogue lacks both the ability to form disulphide bonds and the reducing potential of GSH. Interestingly, when ophthalmic acid (10 mM) was included in the patch pipette, TRPM2 currents were significantly inhibited in TRPM2-HEK293 cells (p < 0.001; Figure 5). This demonstrates that GSH acts in a thiol-independent manner to inhibit TRPM2 currents.

**Figure 6:**
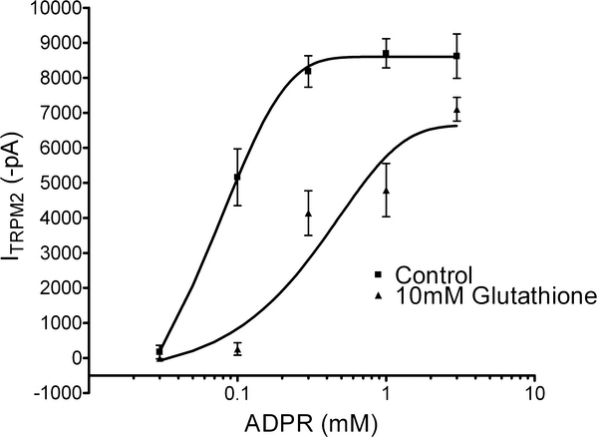
**GSH shifts the ADPR dose-response curve of TRPM2 channels by 3.5 fold**. TRPM2 currents were generated in with or without 10 mM GSH included in the patch pipette. Intracellular concentrations of ADPR used to evoke the current ranged from 0.03 - 3 mM. Each point on the curve represents the mean +/- SE of 6 - 11 recordings. Significant inhibition by GSH was observed for TRPM2 currents evoked by ADPR concentrations of 0.1, 0.3, and 1 mM (*p *< 0.001), with no difference between groups at the highest (3 mM) and lowest (0.03 mM) ADPR concentration points (p > 0.05). The EC_50 _value for ADPR was increased from 77 μM (control) to 269 μM (10 mM GSH) in the presence of 10 mM GSH, representing a ~3.5 fold change in sensitivity to ADPR. Dose-response curves were fit using a sigmoidal dose-response (variable slope), with a Hill slope of 2.2 in control, and 0.9 in the GSH data set.

Potential mechanisms of TRPM2 inhibition by GSH were further studied by constructing a concentration-response relationship for ADPR with or without 10 mM GSH included in the patch pipette. TRPM2 currents were evoked with varying intracellular concentrations (0.03 - 3 mM) of ADPR. Each point on the curve represents the mean +/- SE of 6 - 11 recordings (Figure 6). A two-way ANOVA followed by Bonferroni *post-hoc *test revealed an overall significant effect, with a highly significant inhibition by GSH for TRPM2 currents evoked with ADPR concentrations of 0.1, 0.3, and 1 mM (*p *< 0.001). No difference between groups was observed at the highest (3 mM) and lowest (0.03 mM) ADPR concentrations (p > 0.05). In control conditions, the EC_50 _of ADPR was 77 μM, consistent with a previous report [51]. In contrast, the EC_50 _of ADPR with 10 mM GSH was 269 μM, representing a ~3.5 fold change in sensitivity when GSH is included in the patch pipette. Furthermore, the dose-response curves fit using a sigmoidal dose-response (variable slope), results in a Hill slope of 2.2 in control, and 0.9 in the GSH data set. The rightward shift in the dose-response relationship and decrease in the Hill coefficient confirm that GSH inhibits TRPM2 channels. The results with DTT and ophthalmic acid confirm that the effects of GSH are thiol-independent. However, whether GSH functions as a channel blocker, competes for the ADPR binding site, or interferes with TRPM2 currents by binding to ADPR requires further investigation.

## Discussion

In this study we demonstrate that TRPM2 function increases with time in vitro in cultured hippocampal pyramidal neurons. Moreover, we show that by altering the intracellular concentration of GSH TRPM2 current amplitude can be modulated in this system. Specifically, this was shown in experiments where GSH was supplied exogenously through the patch pipette, or alternatively, where we modulated endogenous GSH metabolism. We also demonstrate that ophthalmic acid, but not DTT, is able to recapitulate GSH mediated inhibition of TRPM2 currents, suggesting that GSH inhibits TRPM2 channels independently of its electron-donating capacity. Lastly, a 3.5-fold shift in the dose-response curve and decrease in the Hill coefficient for ADPR generated TRPM2 currents suggest that GSH either disrupts the co-operative interaction between ADPR and TRPM2 or functions as a channel blocker. Further research is required to determine the precise nature of this interaction. The mechanism through which GSH inhibits TRPM2 and its relevance to the regulation of the channel in neurons is crucial to understand pathways leading to neurotoxicity.

TRPM2 is a Ca^2+ ^permeable non-selective ion channel implicated in oxidative stress-induced cell death [28-30]. Several factors have been reported to modulate TRPM2 current response including Ca^2+ ^concentration, temperature, pH, phosphorylation, the epilepsy related protein EFHC1, and CD38 [28,52,53]. Recently, TRPM2 currents were shown to be inhibited by GSH in sensory neurons [20] as well as in glial cells, where TRPM2 activation following chemically induced GSH depletion contributes to neuroinflammation [20,31]. Our own findings now demonstrate that intrinsic alterations in endogenous GSH content are associated with altered TRPM2 function in hippocampal pyramidal neurons. Collectively, these studies suggest that GSH is a physiologically relevant regulator of TRPM2 and that dynamic changes in GSH content are likely to be associated with parallel changes in TRPM2 function. Importantly, a reduction in GSH has been associated with normal aging in vivo [4-6,8,9]. Neurodegenerative disorders, including Parkinson's disease and progressive supranuclear palsy, are associated with enhanced depletion in intracellular GSH compared to age-matched controls [10-12]. GSH depletion is also observed following ischemia in animal models and in human subjects [13-16]. Diminished levels of reduced GSH has also been linked to bipolar disorder, major depressive disorder, and schizophrenia [54,55]. Importantly, upregulation of glutathione with NAC treatment protects against neuronal cell death in vitro and in vivo [40,56-61].

The findings in this manuscript raise the intriguing possibility that GSH depletion associated with aging, ischemia, and several psychiatric and neurodegenerative disorders, may be associated with enhanced TRPM2 channel function. Future experiments will be required to assess whether TRPM2 currents are also enhanced with age in vivo, and/or in models of neurological disease associated with GSH depletion. Furthermore, the contribution of TRPM2 to neurodegeneration and dysfunction associated with these conditions should be established. Interestingly, depletion of GSH in Parkinson's is one of the earliest biochemical markers of impending neuronal degeneration [12]. Moreover, TRPM2 is expressed and functional in dopaminergic neurons of the substantia nigra [62]. Accordingly, whether TRPM2 currents are enhanced in models of Parkinson's disease (or other neurodegenerative disease in which GSH is depleted) and whether knock-out of TRPM2 may attenuate neurodegeneration has yet to be investigated. Importantly, neurodegenerative diseases are well known to be associated with pronounced neuroinflammatory response. Accordingly, the demonstration that GSH depletion in glial cells promotes neuroinflammation through a mechanism involving TRPM2 [31], paired with our demonstration that GSH depletion facilitates TRPM2 function in vulnerable hippocampal pyramidal neurons, suggests that TRPM2 activation contributes to neuronal demise in neurodegenerative disease through multiple mechanisms. By extension, TRPM2 may represent an important target for the development of novel therapeutic agents of benefit in the treatment of these debilitating neurological diseases.

## Conclusions

The results presented here indicate that GSH plays a physiologically relevant role in the regulation of TRPM2 currents. The effects of GSH are thiol-independent and cannot be recapitulated by reducing agents. Diminished levels of reduced GSH may contribute to calcium dysregulation through the loss of the TRPM2 tonic brake. Therefore, this interaction may play an important role in aging and neurological diseases associated with depletion of GSH.

## Methods

### Reagents

Trypsin-EDTA, 5-fluorodeoxyuridine, uridine, CsOH, Gluconic acid, MgCl2, CaCl2, BaCl_2_, EGTA, KCl, Mg-ATP, N-methyl-D-aspartate (NMDA), D-serine, Glucose, Glutathione (GSH), L-Buthionine sulfoximine (L-BSO), and N-acetyl-cysteine (NAC) were all purchased from Sigma (Oakville, Ontario, Canada). HEPES and NaCl were purchased from Bioshop (Burlington, Ontario, Canada). Ophthalmic acid was obtained from Bachem (Bubendorf, Switzerland).

### Cell culture

Mouse hippocampal primary neuronal cultures were prepared from CD-1 mice (Charles River, Wilmington, MA, USA) at E17-19 as described previously with minor adjustments [63]. Briefly, hippocampal tissue from embryonic day 17-18 mice were dissected, enzymatically and mechanically dissociation with 0.05% Trypsin-EDTA, and plated at a density of < 1 × 10^6 ^cells ml^-1 ^on collagen coated Nunc™ 35 mm culture dishes (Fisher, Rochester, NY). After 3-5 days, each plate was treated for 24 hours with 0.08 mM 5-fluorodeoxyuridine and 0.2 mM uridine. Cells were maintained for 4 weeks in culture.

### Whole-cell recordings: Primary culture

Currents were recorded from mouse hippocampal primary neuronal cultures at 14-28 days in vitro. Whole-cell voltage-clamp recordings were performed as described previously, with minor adjustments [23]. Unless otherwise indicated, standard intracellular solution (ICS) contained (in mM): 150 cesium gluconate, 10 Hepes, and 2 MgCl2 and 1 ADPR. Standard extracellular solution (ECS) contained (in mM): 140 NaCl, 5.4 KCl, 25 Hepes, 33 glucose, 2 CaCl2, and 0.2 TTX. TRPM2 currents were generated with either repeated NMDAR activation or voltage ramps. NMDA currents were elicited with 100 μM NMDA and 3 μM d-serine added to the standard ECS and applied for 5 or 10 seconds every minute using a multibarrelled rapid perfusion system (SF-77B; Warner Instruments, Hamden, CT, USA). Voltage ramps (± 100 mV, 1/10 sec) were applied in the presence of standard ECS supplemented with 1 mM MgCl2 and 1 mM BaCl2. After the TRPM2 current stabilized, or after 10 minutes of recording, calcium free solution was applied (total divalent concentration was maintained by replacing CaCl2 with equimolar BaCl2). Data were filtered at 2 kHz, digitized, and acquired using pCLAMP and Axoscope software (Molecular Devices, Sunnyvale, CA, USA).

### Whole-cell recordings: TRPM2-HEK293

HEK 293 cells stably expressing an inducible flag-tagged human TRPM2 (TRPM2-HEK293) were generously provided by Dr. A. M. Sharenberg (University of Washington, Seattle, Washington). Cells were cultured at 37°C with 5% CO_2 _in DMEM (Sigma) supplemented with 10% fetal bovine serum and 1% penicillin/streptomycin (Invitrogen, Burlington, ON, Canada). TRPM2 expression was induced with doxycycline (1 μg ml^-1^) 24 hours prior to experiments. Intracellular solution (ICS) contained (in mM): 130 CsGluconate, 10 HEPES, 2 MgCl2, 1 CaCl2, 10 EGTA, and 4 Mg-ATP. ICS was adjusted to a pH of 7.3 and osmolarity between 295 and 300 mOsm. The final resistance of ICS-filled electrodes was between 3 and 5 MΩ. The standard extracellular solution (ECS) was composed of (in mM): 140 NaCl, 5.4 KCl, 25 HEPES, 33 glucose, 1 MgCl_2_, 2 CaCl_2_, pH of 7.4 and osmolarity between 310 and 315 mOsm. Calcium-free ECS contained 2 mM BaCl_2 _in the place of CaCl_2_. Data were filtered at 2 kHz, digitized, and acquired using pCLAMP and Axoscope software (Molecular Devices). TRPM2-HEK293 cells were held at -60 mV during whole-cell voltage-clamp recordings. Currents were evoked through the intracellular application of ADPR at a concentration of 0.1 mM unless otherwise indicated. Calcium containing ECS was applied for 5 minutes or until a current of -1000pA developed, followed by 2-3 minutes in calcium-free ECS. Calcium solution was then applied until the TRPM2 current began to inactivate, and the amplitude of the TRPM2 current was then determined after removal of extracellular Ca^2+^.

### Real-time quantitative polymerase chain reaction (QPCR)

Total RNA was extracted from primary mouse hippocampal cultures at 15, 22, and 29 days in vitro (DIV) with Trizol™ (Invitrogen, Burlington, Canada) according to the manufacturer's protocol. RNA concentration was quantified using the Nanodrop Spectrophotometer ND-1000 (Nanodrop technologies Inc., Wilmington, DE, USA). The OD260/280 ratio for all samples was greater than 1.7. Following quantification, RNA was reverse transcribed using Superscript™ II RT reagent according to the manufacturer's guidelines, including the optional treatment with 40 units of RNaseOUT™ (Invitrogen). "No RT" controls contained 1 μL H2O instead of Superscript™ II RT reagent. The reaction was carried out in an Eppendorf Mastercycler personal (Hamburg, Germany).

Primers were designed against the mouse TRPM2 coding strand (accession number NM_138301) and purchased from Sigma. To test primer efficiency, a standard curve was constructed from mouse hippocampal total cDNA using 10x serial dilutions, ranging from 100 ng to 0.1 ng. Inclusion criteria for primers were an efficiency of 90-110%, and an R^2 ^value > 0.98.

To quantify TRPM2 mRNA, 100 ng of cDNA from each sample was run in duplicate with iQ™ SYBR green Supermix (Biorad, Mississauga, Ontario) according to the manufacturer's instructions. The BioRad MyiQTM iCycler was used for real-time qPCR. Negative controls included the No RT samples from reverse transcription and a no-template control containing water instead of cDNA. Biorad MyIQ™ 2.0 software was used for quantification of cycle threshold. TRPM2 primers produced a 90 bp amplicon using the sense sequence 5'TGATCCTGATGGCTGTGGAC3' in combination with antisense sequence 5'AAGAGCAGAATGGCCCCA3'. The reaction was carried out at 95°C for 3 minutes, followed by 40 cycles of 95°C for 1 minute and 55°C for 30 seconds. To control for potential variation in the number of neurons present in culture, cycle thresholds for TRPM2 mRNA were normalized to neuron specific enolase (ENO2) using the sense primer sequence 5'CTGCCTCTGAGTTTTACCGC3' and the antisense primer sequence 5'TCCGGACAAAGTCCTGGTAG3'. ENO2 was quantified with a thermal profile of 95°C for 3 minutes, followed by 40 cycles of 95°C for 1 minute and 62°C for 30 seconds. A a dissociation curve was run for both primers using the following thermal profile: 95°C for 1 minute, 55°C for 1 minute, followed by a 2°C steps every 10 seconds from 70°C to 95°C.

### Statistics

Data are expressed as mean ± SEM. Statistical analysis was undertaken using GraphPad Prism^® ^(GraphPad Software, San Diego, CA). An unpaired two-tailed *t*-test, a one-way ANOVA followed by a Tukey's HSD post-hoc test, or a two-way ANOVA followed by Bonferroni post-hoc test was used to assess statistical significance. Data was considered significant when p < 0.05.

## Abbreviations

GSH: Glutathione; TRPM2: Transient receptor potential melastatin type 2; ROS/RNS: reactive oxygen and nitrogen species; ADPR: adenosine diphosphate ribose; NMDA: N-methyl-D-aspartate; DIV: days in vitro; QPCR: real-time quantitative polymerase chain reaction; ENO2: neuron specific enolase; CT: cycle threshold; NAC: N-acetyl-cysteine; L-BSO: L-buthionine-(S, R)-sulfoximine; DTT: dithiothreitol; ECS: extracellular solution; ICS: intracellular solution.

## Competing interests

The authors declare that they have no competing interests.

## Authors' contributions

JBC designed and performed all experiments. LJG assisted with real-time quantitative PCR analysis. JFM, MFJ, and YFX conceptualized and supervised the project and contributed to the design of experiments. JBC, JFM and MFJ wrote the manuscript. All authors read and approved the final manuscript.
